# Bioprinted Hydrogels for Fibrosis and Wound Healing: Treatment and Modeling

**DOI:** 10.3390/gels9010019

**Published:** 2022-12-27

**Authors:** Jason L. Guo, Michael T. Longaker

**Affiliations:** Department of Surgery, Division of Plastic and Reconstructive Surgery, Stanford University School of Medicine, Stanford, CA 94305, USA

**Keywords:** bioprinting, 3D printing, hydrogels, wound healing, fibrosis, heart, lungs, skin, liver

## Abstract

Three-dimensional (3D) printing has been used to fabricate biomaterial scaffolds with finely controlled physical architecture and user-defined patterning of biological ligands. Excitingly, recent advances in bioprinting have enabled the development of highly biomimetic hydrogels for the treatment of fibrosis and the promotion of wound healing. Bioprinted hydrogels offer more accurate spatial recapitulation of the biochemical and biophysical cues that inhibit fibrosis and promote tissue regeneration, augmenting the therapeutic potential of hydrogel-based therapies. Accordingly, bioprinted hydrogels have been used for the treatment of fibrosis in a diverse array of tissues and organs, including the skin, heart, and endometrium. Furthermore, bioprinted hydrogels have been utilized for the healing of both acute and chronic wounds, which present unique biological microenvironments. In addition to these therapeutic applications, hydrogel bioprinting has been used to generate in vitro models of fibrosis in a variety of soft tissues such as the skin, heart, and liver, enabling high-throughput drug screening and tissue analysis at relatively low cost. As biological research begins to uncover the spatial biological features that underlie fibrosis and wound healing, bioprinting offers a powerful toolkit to recapitulate spatially defined pro-regenerative and anti-fibrotic cues for an array of translational applications.

## 1. Introduction

Bioprinting is a subfield of three-dimensional (3D) printing that utilizes a combination of biomaterials, cells, and/or biophysical factors to produce biologically relevant constructs. Hydrogels have been widely utilized in bioprinting strategies given their ease of adaptability to common 3D printing techniques such as extrusion printing, stereolithography, and more. One critical advantage of hydrogel bioprinting, compared to traditional scaffold fabrication techniques, is that bioprinting enables precise control over both construct architecture and spatial patterning of individual bioink formulations, which can include tissue-specific cellular and/or biochemical components. Thus, hydrogel bioprinting demonstrates a unique potential to replicate the native spatial organization of biological tissues for both therapeutic applications and in vitro modeling.

One promising avenue for 3D hydrogel bioprinting is in the treatment and study of fibrotic diseases, which are spatially complex and often involve the coordinated action of multiple cell populations and tissue types. For instance, pathological skin scarring and regenerative wound healing, which are considered opposing responses to skin injury, are often differentiated by an interplay of multiple biological factors including angiogenesis, fibroblast proliferation, and adipocyte activity [1]. Furthermore, each of these biological processes is dependent on complex interactions between cells, biological ligands, and extracellular matrix (ECM) structures at multiple length-scales [1,2]. Thus, both anti-fibrotic therapies and in vitro models of fibrosis can ideally incorporate multiple cellular or biomolecular agents with biologically relevant spatial organization. Non-bioprinted hydrogel constructs have been used to reproduce the hierarchical properties of wound healing and fibrosis, often through the combination of hydrogel and/or scaffold layers with distinct mechanical properties, biochemical composition, and/or biological functionality [3,4,5]. Additionally, composite constructs have been utilized to provide secondary benefits to tissue regeneration such as anti-microbial treatment during skin wound healing [3,4]. Nevertheless, bioprinting technologies may offer greater control over the distribution of biological and biophysical factors within individual constructs. This review summarizes emerging applications of hydrogel bioprinting technology to wound healing and fibrosis, specifically highlighting how 3D printing techniques have been used to recapitulate the spatial features of native tissues and their associated biological processes.

## 2. Bioprinting Methodologies

Hydrogels can be bioprinted using a wide variety of fabrication techniques, and existing reviews have summarized the breadth of 3D printing technologies compatible with hydrogel-based constructs [6,7,8,9]. An overview of common 3D printing techniques used for hydrogel bioprinting has been provided for reference (Table 1). Among these techniques, extrusion printing may be the most commonly applied modality for hydrogels. Extrusion printing utilizes pneumatic or mechanical force to deposit the bioink from printhead to platform, and can be readily applied to hydrogel bioinks due to its similarity to common extrusion of hydrogels from a syringe [7]. To reduce the imposed shear force on bioinks and/or pressure required for extrusion, hydrogels can be formulated using shear thinning materials, modulated in temperature to reduce viscosity, or deposited as low-viscosity monomeric solutions for crosslinking after printing [7,10]. Stereolithography is another commonly applied technique for hydrogel printing and involves the use of a photocrosslinkable resin in combination with a projected light source to cure the resin within specified X/Y coordinates [11]. This typically operates in a layer-by-layer process, in which horizontal layers of the resin are sequentially cured into specific geometries to collectively produce the user-defined 3D structure [11]. Inkjet printing, in contrast, involves the piezoelectric and/or thermal deposition of small droplets of bioink onto a substrate [6,11]. The deposited droplets then spontaneously fuse and/or chemically crosslink to solidify the printed structure [11]. Other 3D printing modalities that have been applied to hydrogels include laser-assisted bioprinting, which utilizes periodic laser excitation to heat a donor substrate and release spatially defined droplets of bioink adsorbed to the substrate, and melt electrowriting, in which an applied voltage is used to generate spatially defined fluid jetting and direct fiber deposition onto the print platform [11,12].

Hydrogel bioprinting can also be performed in combination with the printing of secondary scaffolds or additional hydrogel formulations to generate composite constructs with mixed material and biological properties. This can be highly advantageous when the hydrogel itself presents relatively weak mechanical properties, as a secondary scaffold can provide mechanical support and maintain stability of the printed structure [9]. Similarly, hydrogels can be printed into a temporary support bath to maintain structure until the completion of printing and/or hydrogel crosslinking. Freeform reversible embedding of suspended hydrogels (FRESH), for instance, utilizes a gelatin microparticle support bath to physically support the deposition of soft, biologically relevant materials, including collagen and fibrin, which is followed by removal of the support bath through temperature elevation [13]. Beyond structural support, composite constructs can offer a more biologically relevant mixture of mechanical and biological properties for heterogeneous tissue types such as osteochondral tissue [9]. For instance, a composite construct that pairs a hard polymer/ceramic scaffold with a soft hydrogel bioink can more effectively recapitulate the distribution of soft and hard tissues—e.g., cartilage, blood vessels, and bone—within the osteochondral unit [9,14]. Thus, multiple bioink formulations and scaffold materials are often combined to generate more spatially complex, biomimetic constructs [9,11].

While hydrogel bioprinting has already enabled the fabrication of complex and spatially heterogeneous tissue constructs, a number of challenges remain for these technologies to fully recapitulate the spatial organization of native tissue. Extrusion printing, for instance, has generally been limited to spatial resolutions of 100–200 µm, and ongoing research has focused on improving print resolution to the sub-micron scale in order to achieve more accurate and reproducible fabrication of tissue-like structures [9]. In addition to improving print resolution, tissue engineering research has focused on improving cell viability during and after printing, which can be impacted by factors such as shear stress during extrusion, exposure to cytotoxic crosslinking reagents, and presence of tissue-specific biomolecules [6,9]. To improve cell viability and proliferation after printing, many bioinks therefore contain growth factors and other biological ligands from the tissue of interest [9]. Similarly, an actively ongoing area of research is the optimization of bioink compositions to mimic the biochemical and physical properties of target tissues. For instance, bone-targeted bioinks may include tissue-specific ratios of calcium phosphates and angiogenic factors, while cartilage-targeted bioinks may instead contain chondrogenic factors and angiostatic factors [15]. For hard tissues such as bone, hydrogels alone may not be able to achieve the requisite mechanical properties and long-term durability of native tissue [15]. Thus, researchers are pursuing strategies for mechanical enhancement of hydrogels such as polymeric fiber reinforcement and co-printing of support scaffolds [16]. Lastly, while hydrogel bioprinting has successfully produced small-scale tissue constructs for research and modeling, translation to the clinic will require improvements to the scalability of bioprinting processes, with additional consideration of factors such as oxygen diffusion, structural integrity, and print speed for the accurate production and long-term viability of larger-scale tissue constructs [9,17].

## 3. Treatment of Fibrosis with Bioprinted Hydrogels

Fibrosis is defined as the pathological accumulation of excessive ECM, and can occur in nearly every organ. While collagenous ECM is largely deposited by fibroblasts, multiple cell phenotypes and multicellular communities are responsible for the initiation and sustenance of pro-fibrotic conditions [1]. Thus, the treatment and prevention of fibrosis can be achieved by modulation of a variety of cellular and biochemical processes, including inflammation, pro-fibrotic fibroblast activation, and angiogenesis [1]. Fibrosis is also a spatially complex phenomenon that produces locally defined ECM architectures and microenvironments, which may vary depending on the specific organ and underlying pathology [18,19,20]. Anti-fibrotic therapies may therefore benefit from targeting these unique spatial biological features at the site of fibrosis. Bioprinting technologies can directly address these requirements through the fabrication of patient- and tissue-specific hydrogels with tailored geometry and spatial patterning of cells and biological factors [9]. In this section, we describe the applications of hydrogel bioprinting to treatment of fibrosis in a highly diverse set of organs, including the skin, heart, and endometrium.

### 3.1. Dermal Fibrosis

As skin heals from injury, it often exhibits pro-fibrotic scarring, which is exacerbated by mechanical tension and is characterized by the development of a thickened dermis with highly aligned collagen fibers [1]. While many bioprinted hydrogels have been developed for purposes of skin tissue engineering, a few have been designed specifically to reduce scar formation at the site of injury [11,21,22]. Bioprinted hydrogels for dermal fibrosis treatment have primarily been designed to replicate biological processes and architectural features that distinguish healthy skin from scars, such as angiogenic progression and random, rather than parallel, orientation of collagen fibers [1]. Chen et al., for instance, printed decellularized ECM (dECM) hydrogels that retained the topological and mechanical properties of healthy dermal matrix, which effectively reduced skin contraction and scar formation upon implantation [21]. Ibañez et al., on the other hand, modulated the stiffness and porosity of bioprinted gelatin methacrylate hydrogels to reduce pro-fibrotic activation of dermal fibroblasts [22]. They observed that highly porous, low-stiffness, and angiogenic-factor-releasing hydrogel constructs—which mimicked the architectural, mechanical, and biological properties of healthy dermis—reduced pro-fibrotic gene expression and collagen deposition by dermal fibroblasts [23]. Collectively, these studies demonstrate that biomimicry of healthy skin can help reduce the pro-fibrotic response during skin repair.

### 3.2. Cardiac Fibrosis

**Figure 1:**
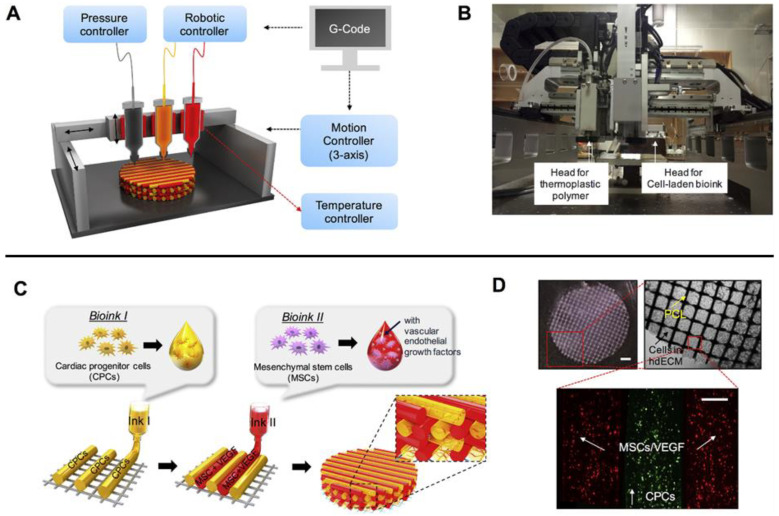
3D printed patch for cardiac fibrosis treatment, with user-defined spatial patterning of cardiac progenitor cells and mesenchymal stem cells. (**A**) Schematic of extrusion-based 3D printing system. (**B**) Photograph of extrusion printer. (**C**) Patterning of distinct bioink formulations and cell types within each layer. (**D**) 3D printed patch, including zoom-ins of layer-based geometry and distribution of cell types within printed fibers. Top-left scale bar represents 1 mm, while bottom scale bar represents 200 μm. Adapted with permission from [27], 2017, Jang et al.

Cardiac fibrosis can accumulate progressively over time, as in the case of age-associated interstitial and perivascular fibrosis, or acutely following injury, as observed in the myocardium following myocardial infarction (MI) [24]. Bioprinted hydrogels for cardiac fibrosis treatment have focused on achieving cardiac tissue regeneration using a variety of cell types such as mesenchymal stem cells (MSCs) and cardiomyocytes, often in tandem with the inhibition of pro-fibrotic processes via controlled release of biochemical ligands. Guan et al., for instance, bioprinted hydrogels that contained endothelial cells and cardiomyocytes for cardiac regeneration, as well as splenic dECM for provision of cardioprotective factors such as IL-10 [25]. Implantation of these hydrogels in a mouse model after MI promoted neovascularization and the return of healthy cardiac function, as well as reduced fibrotic tissue growth [25]. In another example, Melhem et al. used stereolithography to print poly(ethylene glycol) (PEG) hydrogels with patterned microchannels for the delivery of MSC secreted paracrine factors [26]. The authors found that inclusion of the microchannels, which enhanced cytokine diffusion into the native tissue, promoted cardiac function and minimized scar formation [26]. Jang et al., on the other hand, used extrusion printing to generate cardiac dECM hydrogel patches with user-defined spatial patterning of cardiac progenitor cells and MSCs for spatially directed blood vessel formation (Figure 1) [27]. Interestingly, the directed patterning of these cell types resulted in enhanced cardiac muscle regeneration and reduced fibrosis following MI, compared to a spatially uniform distribution of cell types [27]. Ultimately, these studies demonstrate that cardiac fibrosis can be inhibited by modulating a diverse set of biological processes that take place following injury. Furthermore, optimal cardiac regeneration may be achieved by recapitulating the spatial organization of tissue types observed in healthy cardiac tissue.

### 3.3. Intrauterine Adhesions

Intrauterine adhesions (IUAs) are bands of fibrotic ECM that form within the endometrial cavity, and are a common pathological response to uterine injury, inflammation, and/or surgical operation [28]. IUAs can lead to a number of negative consequences related to reproductive health, including miscarriage and infertility [28]. Bioprinted hydrogels for IUA treatment often focus on providing a physical barrier to adhesion formation, and some strategies also incorporate pro-regenerative factors or cell types such as MSCs to heal tissues within the endometrial cavity [29,30,31]. This was exemplified by Feng et al., who used extrusion printing to develop gelatin/collagen hydrogels as a physical barrier to IUA formation following injury in a rat model [29]. Wen et al., in contrast, used poly lactic-coglycolic acid microspheres to solubly deliver granulate colony stimulating factor from a 3D printed gelatin/alginate hydrogel, promoting simultaneous endometrial tissue regeneration and physical-barrier-based inhibition of IUAs [30]. Ji et al. similarly encapsulated MSCs within a gelatin/alginate hydrogel in order to promote endometrial repair [31]. Overall, these applications highlight the ability of 3D printed hydrogel constructs to serve dual functions of inhibiting fibrotic tissue growth while promoting healthy tissue regeneration.

### 3.4. Challenges and Future Directions

Bioprinting offers the potential to develop site-specific constructs that modulate a number of biological processes associated with fibrosis. Nevertheless, most bioprinted hydrogels have incorporated a limited array of cell phenotypes and biological ligands—typically, those relevant to angiogenesis, healthy tissue regeneration, or inhibition of inflammatory processes [8,23,25,30]. Another promising route of investigation may be the targeted modulation of activated fibroblast subpopulations, which are largely responsible for fibrotic ECM deposition [1,24]. For instance, bioprinted hydrogel constructs may be used to deliver ligands that target specific pro-fibrotic and pro-regenerative fibroblast subpopulations identified in recent single-cell RNA sequencing (scRNA-seq) studies of fibrosis [18,19]. Alternatively, given the near-universal relevance of mechanobiological factors to fibrosis in various organs, bioprinted hydrogels may be adapted to target specific mechanotransduction pathways such as FAK and YAP/TAZ [18,32]. Future bioprinting strategies may therefore take inspiration from emerging single-cell biology and mechanobiological studies to more directly target the cellular drivers of fibrosis.

## 4. Wound Healing Using Bioprinted Hydrogels

Skin undergoes sequential and overlapping stages of regeneration following injury [1]. Following acute trauma, the skin undergoes hemostasis, acute inflammation, proliferation, re-epithelialization, ECM deposition, and remodeling [33]. Acute wound healing is often marked by scarring, which is predominantly driven by excessive, pro-fibrotic collagen deposition during the proliferative phase [33]. The resulting scar tissue is poorly functional compared to healthy skin, exhibiting inferior mechanical properties, hair follicle development, gland formation, and more [1]. Thus, hydrogels for acute wound treatment must carefully balance the promotion of skin growth with the inhibition of pro-fibrotic processes. Chronic wounds, in contrast, are characterized by a stark absence of dermal regeneration and often occur as a consequence of prolonged inflammation, oxidative stress, diabetic conditions, necrotic tissue accumulation, pressure injury, or other factors that inhibit progression of the typical wound-healing sequence [33,34]. Thus, acute and chronic wounds exhibit highly distinct microenvironments and present unique design requirements for hydrogel bioprinting.

### 4.1. Acute Wounds

**Figure 2:**
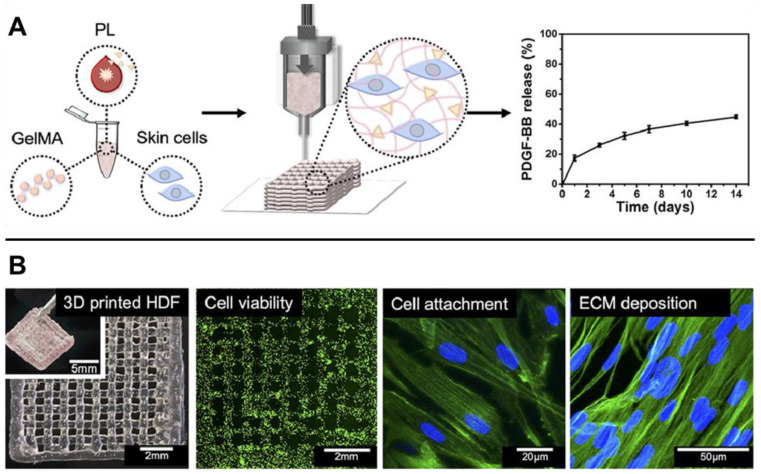
Gelatin methacrylate (GelMA) hydrogel with encapsulated platelet lysate (PL) and human dermal fibroblasts (HDFs) for acute wound healing. (**A**) Schematic of hydrogel bioprinting. Incorporation of PL resulted in sustained release of angiogenic PDGF-BB over 14 days. (**B**) 3D printed constructs were cytocompatible and promoted dermal cell attachment and matrix deposition. Adapted with permission from [38], 2021, Daikuara et al.

Acute wounds can occur from surgery, physical trauma, thermal injury, and other sources of tissue damage [33]. While local cell proliferation and ECM deposition are required for skin regeneration, these processes often must be balanced by inhibition of wound contraction, local inflammation, and other factors that drive fibrotic tissue growth [35]. Hydrogels for acute wound treatment often consist of pro-regenerative moieties, such as skin-derived biopolymers and various angiogenic factors, as well as encapsulated biochemical factors for the inhibition of fibrosis. To address the latter requirement, Navarro et al. printed keratin hydrogels loaded with Halofuginone, an inhibitor of collagen I synthesis, in order to delay the kinetics of ECM deposition and inhibit the onset of scarring [36]. The resulting hydrogels produced superior non-fibrotic wound healing, including gland and hair follicle formation, in a porcine thermal burn model [36]. Yu et al., on the other hand, printed gelatin hydrogels doped with polycaprolactone (PCL) nanofibers, which present low fiber diameter and high porosity that mimic healthy skin and inhibit fibrosis [37]. Given the importance of angiogenesis to wound healing, many bioprinted hydrogels have also incorporated pro-angiogenic factors [33,38,39]. Jang et al., for instance, incorporated vascular endothelial growth factor-mimicking peptides [39], while Daikuara et al. encapsulated platelet lysate within 3D printed gelatin hydrogels (Figure 2) [38]. Overall, bioprinted hydrogels for acute wound treatment often recapitulate specific biological processes associated with scarless wound healing in the native skin.

### 4.2. Chronic Wounds

**Figure 3:**
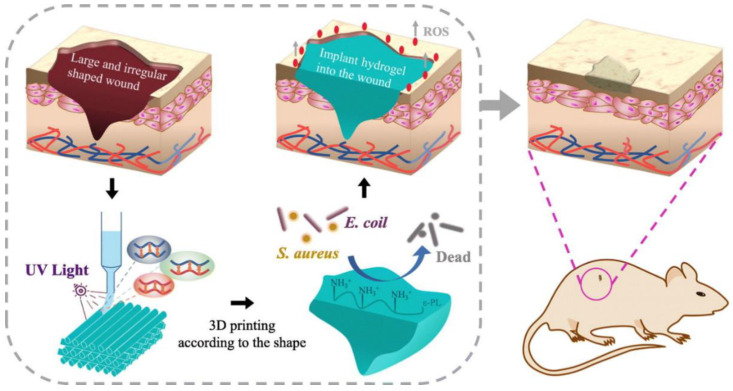
Bioprinting of geometrically complex hydrogel for chronic wound treatment, including conjugated polylysines for antibacterial effects and reduction of reactive oxygen species (ROS) at the wound site. Adapted with permission from [42], 2021, Wang et al.

Chronic wounds are frequently associated with vascular disease and/or diabetes, and are characterized by a number of common biological features such as prolonged inflammation, susceptibility to infection, and accumulation of reactive oxygen species (ROS) [34]. Bioprinted hydrogels for chronic wound treatment are therefore often designed as multifunctional constructs with regenerative, antibacterial, and/or antioxidant capabilities. In one example of antibacterial treatment, Cleetus et al. 3D printed alginate hydrogels with doped ZnO nanoparticles, which decreased bacterial growth but did not affect mammalian cell viability [40]. Wu et al. similarly incorporated silver nanoparticles in bioprinted polyacrylamide hydrogels, which produced superior healing in *Staphylococcus aureus*-infected chronic wounds [41]. Other chronic-wound-targeted hydrogels have included ROS-scavenging components to help reduce oxidative stress. Wang et al., for example, printed carboxymethyl cellulose hydrogels with covalently conjugated polylysine moieties for both antibacterial effects and ROS reduction, which improved tissue regeneration in a rat model of chronic wounds (Figure 3) [42]. Thus, bioprinted hydrogels for chronic wound treatment have benefited from the incorporation of secondary antibacterial and antioxidant functionalities, which may help ameliorate some of the underlying pathological factors involved in the persistence of chronic wounds [34].

### 4.3. Challenges and Future Directions

**Figure 4:**
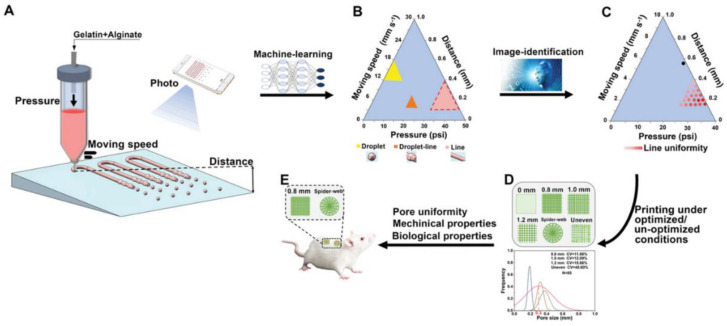
Machine-learning-based optimization of scaffold design and 3D printing parameters for wound healing. (**A**) Schematic of extrusion printing and layer-based photo acquisition. (**B**) Modeling of printed morphology based on input parameters. (**C**) Identification of input parameters for desired morphologies. (**D**) Generation of variable layer geometries using optimized printing conditions. (**E**) Association of macroscopic hydrogel properties with biological outcomes in wound healing. Adapted with permission from [43], 2022, Chen et al.

Bioprinted hydrogels have successfully addressed many of the unique biological requirements of acute and chronic wound treatment, often through the development of multifunctional hydrogels. However, the optimization of hydrogel design and 3D printing parameters can be a highly time-consuming process, particularly for geometrically complex chronic wounds. Emerging strategies have therefore utilized machine learning (ML) to accelerate the construct-design process [43,44,45]. In one promising example, Chen et al. utilized deep learning for high-throughput screening and selection of alginate-gelatin printing parameters for diabetic wound healing (Figure 4) [43]. Using this ML-driven approach, the authors identified an optimal scaffold design and associated print parameters that maximized tissue regeneration both in vitro and in vivo [43]. Nevertheless, ML analysis has not yet been widely adopted in bioprinting research [45]. Additionally, most bioprinted hydrogels for wound treatment, similar to those designed for anti-fibrotic therapy, have utilized a relatively limited array of cell phenotypes and biological ligands. Future research may be able to build upon recent scRNA-seq analyses of acute and chronic wounds to target more specific cell subpopulations, including transcriptionally defined fibroblast subtypes, that modulate these unique wound microenvironments [1,18,19].

## 5. In Vitro Modeling of Fibrosis Using Bioprinted Hydrogels

Hydrogel bioprinting has been used to produce cell- and organoid-based systems for the in vitro modeling of fibrotic disease, which can offer a high-throughput method to screen anti-fibrotic drug treatments at a relatively lower cost compared to animal models [46]. Compared to animal models, these in vitro bioprinted models also offer finer control over microenvironmental factors such as biochemical composition, mechanical loading, and cell phenotypic ratio [46,47]. Thus, bioprinted tissue models can be utilized to isolate the individual mechanistic factors that contribute to fibrotic progression. For instance, the effects of fibrotic drug treatment on individual cell types of interest, such as activated fibroblasts, can be studied in isolation using bioprinted models [46]. Fibrotic disease progression has been modeled using hydrogel bioprinting in a number of biologically distinct tissues, including the skin, heart, liver, lungs, and muscle [48,49,50,51,52]. In this section, we specifically highlight applications in fibrotic disease modeling and drug screening for the most commonly studied tissue types in this area of research: the skin, heart, and liver.

### 5.1. Dermal Models

Skin is a heterogeneous and multilayered organ, with variations in cellular and biochemical composition from the subdermal to dermal and epidermal layers [53]. Many bioprinting approaches to mimicking the skin have therefore featured sequential deposition of layer-specific cell types such as fibroblasts and keratinocytes for the dermis and epidermis, respectively [11,48,54]. For example, Lee et al. deposited alternating layers of collagen, fibroblasts, and keratinocytes to produce 3D constructs that mimicked the morphological properties of native skin [48]. Kim et al., instead, printed fibroblast- and keratinocyte-loaded bioinks within a transient gelatin support bath and PCL scaffold to maintain the structural stability of their skin construct during printing [54]. In another example, Bin et al. developed in vitro models of scar tissue using extrusion printing of pre-cultured aggregates of scar-derived fibroblasts and scar-derived dECM [55]. As a proof-of-concept for drug screening, the authors tested two anti-fibrotic drugs, Abemaciclib and Cobimetinib, and observed a corresponding reduction in pro-fibrotic gene expression [55]. Thus, bioprinting can be used to generate multilayered and biologically representative models of skin for both drug screening and fundamental scientific studies.

### 5.2. Cardiac Models

MI generates complex changes in cellular and biochemical composition, and a number of in vitro models have been bioprinted to mimic the spatially restricted growth of fibrotic tissue following infarction. The compressive modulus of myocardial tissue, for instance, increases by approximately an order of magnitude as a result of post-MI cardiac fibrosis [24]. Accordingly, Shin et al. developed an extrudable bioink of cardiac dECM, laponite, and PEG that could produce hydrogel constructs with regionally defined compressive moduli of 13.4–89 kPa by the variation of PEG concentration [49]. In another example, Daly et al. modulated the relative concentrations of cardiomyocytes and cardiac fibroblasts in distinct areas of a 3D printed, spheroid-based microtissue to mimic the localized onset of fibrotic regions [56]. The authors then utilized their tissue-engineered model to study the influence of microRNA therapeutics on cardiac tissue repair [56]. In addition to drug screening, 3D printed cardiac models have been developed for surgical planning and education. For example, Valverde et al. used patient-derived computed tomography scans to print anatomical constructs of the heart using fused deposition modeling, which were used for patient-specific surgical planning [57]. Overall, bioprinted models of cardiac fibrosis have been used for a diverse set of applications in fibrotic drug screening, scientific analysis, and augmentation of clinical practice.

### 5.3. Liver Models

**Figure 5:**
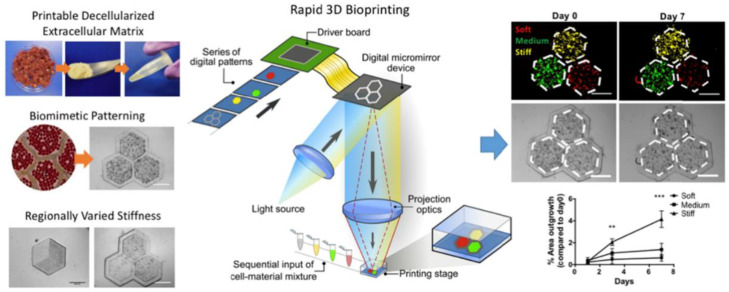
3D printing of decellularized liver matrix with biomimetic morphology and user-defined patterning of regional stiffness. Greater stiffness resulted in enhanced tumor cell invasion into surrounding regions (bottom right). Adapted with permission from [60], 2018, Ma et al.

Hepatotoxicity screening is a critical component of the drug development process, and fibrosis is one of the most common outcomes of drug-induced liver injury [58]. Thus, in vitro models that can recapitulate the liver’s response to chemical injury, including fibrogenesis, have immense value for academic and industrial research [58]. In one notable example, Norona et al. bioprinted 3D liver tissues consisting of hepatocytes, hepatic stellate cells, and endothelial cells, which underwent hepatocellular damage and progressive fibrogenesis in response to methotrexate and thioacetamide exposure [50]. In another example, Nguyen et al. extrusion-printed liver models using a similar composition of cell phenotypes, producing 3D tissues that outperformed 2D cell-culture models in replicating native intercellular hepatocyte junctions and endothelial networks [59]. Interestingly, their 3D printed tissues exhibited dose-dependent toxicity in response to clinically relevant concentrations of Trovafloxacin, supporting the translational utility of their bioprinted construct [59]. In addition to modeling drug-induced liver fibrosis, bioprinted hydrogels have been used to mimic the progression of fibrosis associated with hepatic cancers. Ma et al., for instance, used digital light processing to print liver dECM hydrogels that promoted variable degrees of hepatocellular carcinoma cell proliferation, invasion, and metastatic gene expression depending on regional material stiffness (Figure 5) [60]. As shown by these examples, bioprinting enables user-defined spatial control of the biological and biophysical parameters that influence fibrotic processes in the liver.

### 5.4. Challenges and Future Directions

Hydrogel bioprinting has produced next-generation in vitro models of fibrosis in the skin, heart, liver, and other tissues. Nevertheless, many architecturally complex organs continue to present technical difficulties for model fabrication. For instance, the lungs contain highly branched and mutually entangled networks of vascular and pulmonary vessels that are difficult to replicate in vitro, particularly for soft materials that are highly biomimetic but may present poor structural integrity during printing [9]. Recent advances in 3D printing technology, such as the adoption of cytocompatible photoabsorbers to spatially constrain light-induced polymerization, may be able to address this by enabling more geometrically complex architectures to be printed using stereolithography and other light-based techniques [9,61]. Additionally, the development of biomimetic tissue models requires a granular map of 3D cell phenotypic organization in the native tissue. The recent emergence of RNA- and protein-based spatial mapping technologies such as Visium and CO-Detection by indEXing (CODEX) may support this by enabling spatial phenotyping of intact tissue sections at single-cell spatial resolution [62]. Nevertheless, these new spatial datasets must be translated from 2D to 3D in order to produce compatible blueprints for hydrogel bioprinting.

## 6. Conclusions

Hydrogel bioprinting, while a relatively new field, has already enabled the development of increasingly complex and biomimetic constructs for the treatment of fibrosis, promotion of wound healing, and in vitro modeling of native biological processes. Emerging applications of hydrogel printing include the creation of site-specific constructs for anti-fibrotic therapy, multifunctional hydrogels for acute and chronic wounds, 3D tissue models for fibrotic drug screening, and more. Excitingly, bioprinting technology has been used to recapitulate the spatial organization of cells and biochemical factors in native tissue, replicating pro-regenerative biological moieties at high resolution. Future research can build upon these advances in hydrogel bioprinting by targeting and/or recapitulating more granular cell subpopulations that drive fibrosis and wound healing, as identified in recent scRNA-seq and spatial sequencing studies.

## Figures and Tables

**Table 1:** Common 3D printing techniques utilized for hydrogel bioprinting.

3D Printing Technique	Common Advantages	Common Limitations
Extrusion printing	+ Facile printing process and setup + Compatibility with many common hydrogel materials + Precise control of individual printheads/bioink conditions	- Lower resolution than some other techniques - Shear stress on bioink components - Potential for printhead clogging - Difficulties in printing overhanging parts
Stereolithography	+ Very high resolution + Ability to achieve complex architectures + High consistency enabled by control of light source settings	- Requirement for photocrosslinkable material - Potential cytotoxicity of reagents - Greater difficulty in achieving horizontal gradients
Inkjet printing	+ High speed of printing + Low cost + Precise control of individual printheads/bioink conditions	- Requirement for more specific, low-viscosity materials - Potential cytotoxicity of piezoelectric or thermal conditions - Potential for printhead clogging
Laser-assisted bioprinting	+ High resolution + Precise horizontal patterning of cells and/or biomolecules + Cytocompatible conditions due to absorption of laser by donor substrate	- High cost - Requirement for specific bioinks adsorbable to donor substrate - Limitations in scale of printed construct
Melt electrowriting	+ Ability to produce highly porous constructs and thin fibers + Replication of fibrillar structures found in native ECM + Low cost	- More extensive trial-and-error in determining printing parameters - Greater susceptibility to environmental conditions - Less predictability in achieving precise fiber deposition
